# Genome sequence of *Kobresia littledalei*, the first chromosome-level genome in the family Cyperaceae

**DOI:** 10.1038/s41597-020-0518-3

**Published:** 2020-06-11

**Authors:** Muyou Can, Wei Wei, Hailing Zi, Magaweng Bai, Yunfei Liu, Dan Gao, Dengqunpei Tu, Yuhong Bao, Li Wang, Shaofeng Chen, Xing Zhao, Guangpeng Qu

**Affiliations:** 1 State Key Laboratory of Hulless Barley and Yak Germplasm Resources and Genetic Improvement, Lhasa, 850000 China; 2 Institute of Grassland Science, Tibet Academy of Agriculture and Animal Husbandry Science, Lhasa, 850000 China; 3 Novogene Bioinformatics Institute, Beijing, 100083 China

**Keywords:** Genomic analysis, Plant sciences, DNA sequencing, Genome

## Abstract

*Kobresia* plants are important forage resources in the Qinghai-Tibet Plateau and are essential in maintaining the ecological balance of grasslands. Therefore, it is beneficial to obtain *Kobresia* genome resources and study the adaptive characteristics of *Kobresia* plants in the Qinghai-Tibetan Plateau. We assembled the genome of *Kobresia littledalei* C. B. Clarke, which was about 373.85 Mb in size. 96.82% of the bases were attached to 29 pseudo-chromosomes, combining PacBio, Illumina and Hi-C sequencing data. Additional investigation of the annotation identified 23,136 protein-coding genes. 98.95% of these were functionally annotated. According to phylogenetic analysis*, K. littledalei* in Cyperaceae separated from Poaceae about 97.6 million years ago after separating from *Ananas comosus* in Bromeliaceae about 114.3mya. For *K. littledalei*, we identified a high-quality genome at the chromosome level. This is the first time a reference genome has been established for a species of Cyperaceae. This genome will help additional studies focusing on the processes of plant adaptation to environments with high altitude and cold weather.

## Abstract (summary)

**Table:** 

Measurement(s)	genome • DNA • sequence_assembly • sequence feature annotation
Technology Type(s)	DNA sequencing • sequence assembly process • sequence annotation
Factor Type(s)	sequencing platform
Sample Characteristic - Organism	Carex littledalei
Sample Characteristic - Location	Tibetan Plateau

Machine-accessible metadata file describing the reported data: 10.6084/m9.figshare.12326612

## Background & summary

**Fig. 1:**
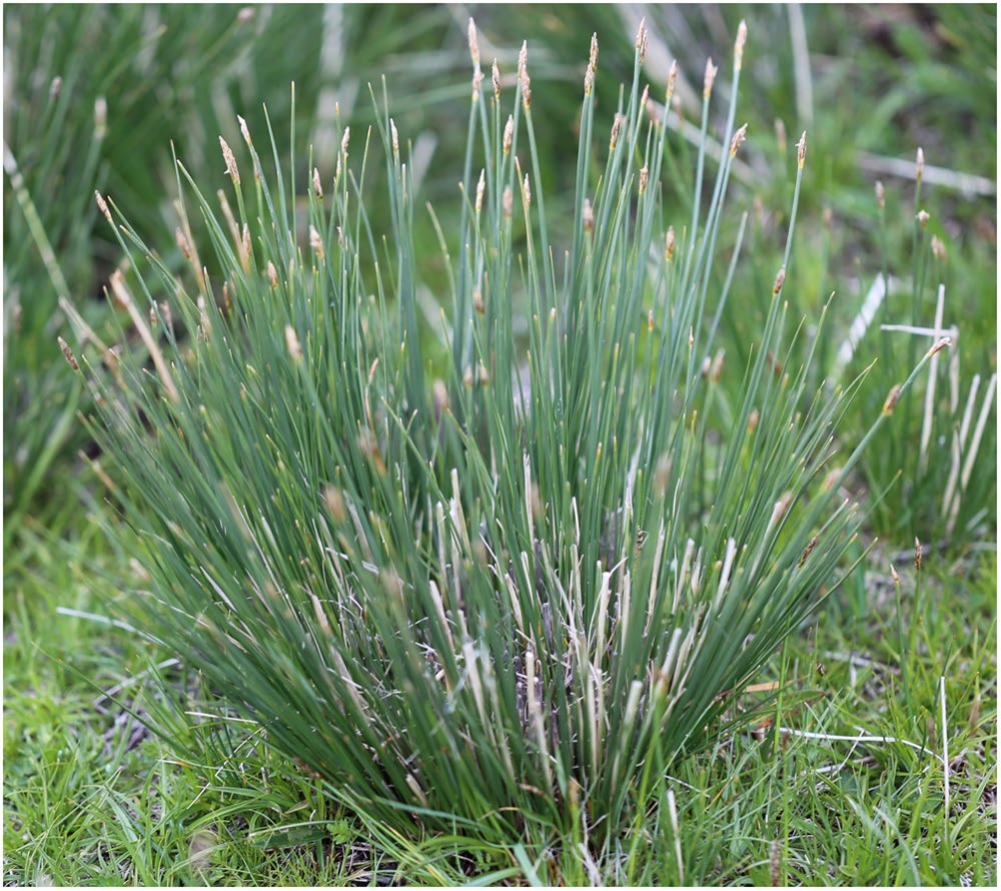
A representative individual of *Kobresia littledalei*.

The Qinghai-Tibet Plateau, known as the “roof of the world”, is a vast alpine steppe with harsh natural conditions of high altitude, cold, intense ultraviolet radiation and drought. After a long period of natural selection, most of the forage germplasm resources in this area have desirable genes such as resistance to cold and drought, which are indispensable materials for breeding improved varieties of crop plants. Tibet’s natural grasslands are rich in wild forage germplasm resources, and *Kobresia* plants (Cyperaceae) are the most important component of these alpine grasslands. *Kobresia* plants are perennial herbs that are mainly distributed in temperate to cold zones of the Northern Hemisphere and are mostly concentrated in the Himalayas and Hengduan Mountains. *Kobresia* plants are important forage resources in the Qinghai-Tibet Plateau due to their nutritious features. In addition, *Kobresia* plants are essential in maintaining the ecological balance of grasslands because they are tolerant of cold, radiation, drought and strong wind. *K. littledalei* are mainly distributed in low-lying areas along the edge of lakes and rivers and are used as mowed grasslands and winter grazing grasslands (Fig. 1).

The *K. littledalei* genome was assembled and annotated using long reads obtained from the PacBio Sequel sequencing program and short reads from the Illumina Hi-seq. 2500 sequencing program. We determined that the final genome assembly has a contig N50 of ~2.55 Mb and is approximately 373.85 Mb. Using Hi-C data, we determined that 96.28% of the assembled bases were associated with 29 pseudo-chromosomes. *K. littledalei* represents the first assembled genome in Cyperaceae. We identified 23,136 protein-coding genes from the generated assembly, annotating 98.95% (22,892 genes) of all the protein-coding genes. We determined that *K. littledalei* separated from Poaceae about 97.6 million years ago after separating from Bromeliaceae about 114.3 million years ago. The genome assembly of *K. littledalei* provides an important framework for the additional study of adaption to environment of high altitude and cold weather and promote the protection of the environment in the Qinghai-Tibet Plateau.

In Poales, genomes of some species in Poaceae and one species in Bromeliaceae have been sequenced and assembled. Cyperaceae is more closely related to Poaceae than Bromeliaceae^1^, and the assembly of the genome *K. littledalei* offers an opportunity for the investigation of Poales evolution.

## Methods

### Sample sequencing and genome size estimation

High-quality genomic DNA for sequencing was extracted from leaf tissue of *K. littledalei*, which was collected in July 2018 from DangXiong in the Tibet Autonomous Region of China. The sample was in anthesis, and located at altitudes of up to 4,263 m.

The Illumina library with insert sizes of 350 bp was arranged with a Genomic DNA Sample Preparation kit from Illumina. It was then sequenced using a HiSeq 2500 platform, also from Illumina. This yielded 168.79 million reads, ∼50.64 Gb of raw sequence data, which covered ~121.95X of the genome (Table s1). Large DNA fragments longer than 10 kb were enriched and were then sequenced using a PacBio Sequel system. From this, we obtained 5,618,892 reads that had an N50 length of 17,273 base pairs and a mean of 11,099 base pairs. In total, 62.37G bases were obtained, which is ~150.20X coverage of the genome (Table s1). Leaf tissue of *K. littledalei* was used to construct a library for Hi-C analysis, and the NEBNext Ultra II DNA library Prep Kit from Illumina (NEB) was used to prepare the Hi-C library, which we then sequenced using the Illumina HiSeq X Ten platform. 230,316,080 paired-end reads of 150 bp were obtained from the Illumina sequencing platform for the Hi-C library, which covered ~166.40X of the genome (Table s1).

The size and heterozygosity level of the *K. littledalei* genome were estimated through k-mer spectrum analysis using sequences generated by Illumina DNA sequencing technology^2^. The depth distribution of the derived 17-mers clearly showed two separate peaks and the main volume peak of k-mer frequency was 96, based on which we estimated the heterozygosity level and repeat frequencies of the *K. littledalei* genome to be 1.68% and 53.93%, respectively; the genome size was estimated to be 415.24 Mb (Fig. s1, Table s2).

### Assembly of the *Kobresia littledalei* C. B. Clarke genome

First, PacBio long reads were self-corrected to obtain pre-assembly reads. The pre-assembly reads were assembled into consensus sequences by FALCON through the “Overlap-Layout-Consensus” algorithm^3^. Consensus sequences were corrected using Illumina short reads to improve the precision in Pilon^4^. The preliminary genome assembly of *K. littledalei* includes 1210 contigs with N50 = 2,253,412 bp and longest scaffold = 11,050,451 bp. The genome is approximately 759 M in length and the GC content of the genome is 35.74% (Table s3).

Purge Haplotigs was used to filter redundant sequences due to heterozygosity^5^. The final assembled *K. littledalei* genome contained 212 scaffolds with an N50 length of 3,054,069 bp and a cumulative size of 373,821,983 bp. The longest scaffold reached 11,045,779 bp, and the GC content of the genome was 35.44% (Table 1)

**Table 1:** Statistics of assembled *Kobresia littledalei* C. B. Clarke assembly and annotation.

Genome Assembly	
Number of scaffolds	212
Total length of scaffolds (bp)	373,821,983
N50 of scaffolds (bp)	3,054,069
Longest scaffold (bp)	11,045,779
GC content (%)	35.44
Genome Assembly (Hi-C version)	
Number of scaffolds	523
Total length of scaffolds (bp)	373,852,675
N50 of scaffolds (bp)	2,548,827
Longest scaffold (bp)	7,550,132
GC content (%)	35.44%
Repeat annotation	
Total (bp)	202,340,678
TRF (bp)	20,963,545
Transposable element (bp)	197,921,429
Gene annotation	
Number of genes	23,076
Total coding sequence length (bp)	81,810,189
Mean gene length (bp)	3545.25
Mean number of exons per gene	5.39
Mean exon length (bp)	215.84
Average CDS length (bp)	1163.41

We used the procedures described by DC. Zhang *et al*.^6^ to anchor the scaffolds into pseudo-chromosomes. We first used HiCUP v0.6.1^7^ to map and process reads obtained from the Hi-C library. Each of the reads from one pair were uniquely mapped to the assembly and kept for downstream filtration. Invalid pairs generated from fragments of the wrong size, PCR duplication, re-ligation, internal fragments, dangling ends, circularization, and contiguous sequences were removed. *K. littledalei* has 2n = 58 chromosomes, as determined by karyotype analyses. We corrected some small errors in the results of the FALCON assembly by clustering contigs with the contig contact frequency matrix. We obtained 523 total contigs by grouping contigs with errors into shorter contigs. Using Lachesis v1.0^8^ (pseudo-chromosome number set as 29), we clustered 337 contigs into pseudo-chromosomes using the refined alignments. This corresponds to 96.28% of the assembly by base count and 64.44% of the assembly by sequence number.

**Fig. 2:**
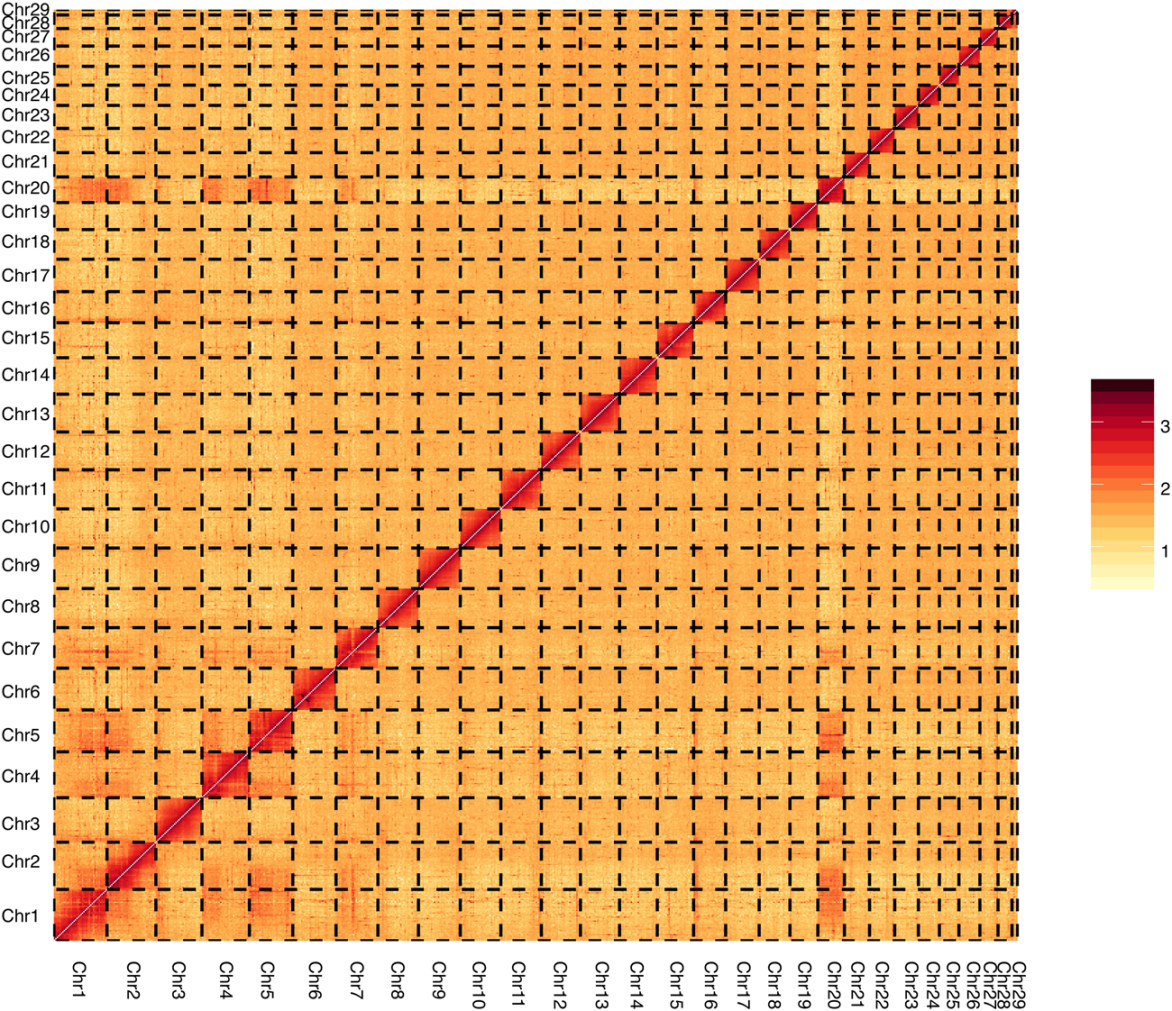
Heat map of chromatin contact matrices generated by aligning a Hi-C dataset to the *Kobresia littledalei* genome. The frequency of interactions was calculated using a window size of 500 K.

We built an interaction matrix by HiC-Pro using clean reads from the Hi-C library to confirm the accuracy of the Hi-C scaffolding at the pseudo-chromosome level^9^. The genome was split into equal-sized bins of 500k, while the contact numbers were designated between each pair of bins. We confirmed the genome quality and structure using a contact map plotted with HiCPlotter^10^ (Fig. 2).

### Repeat annotation

We used RepeatMasker^11^ to predict repeat sequences of the *K. littledalei* genome through homology searching of repetitive elements released by Repbase^12^ and *ab initio* identified by LTR Finder^13^, RepeatScout^14^ and RepeatModeler. We identified a total of ~202.34 M repetitive elements, which was 54.13% of the genome, after integrating ~40.04 M repetitive elements predicted by RepeatProteinMask and ~20.96 M tandem repetitive sequences predicted by TRF^15^ (Table s4). Among them, DNA transposons accounted for 18.53% of the genome, while long terminal repeat (LTR), long interspersed nuclear elements (LINE) and short interspersed nuclear elements (SINE) belonging to retrotransposons accounted for 27.87%, 5.42% and 0.10% of the genome respectively (Table s5).

### Prediction and functional annotation of protein-coding genes

The repeat-masked *K. littledalei* C. B. Clarke genome was used for subsequent prediction of protein-coding genes, which integrates evidence from *de novo* predictions, protein homology and RNA transcripts. Augustus^16^, GlimmerHMM^17^, SNAP^18^, Geneid^19^ and Genscan^20^ were used for *ab initio* gene prediction. For homolog searches, we used proteomes of *Zea mays*, *Brachypodium distachyon*, *Oryza sativa*, *Setaria italica*, *Ananas comosus* and *Arabidopsis thaliana*. Due to the lack of RNA-seq data of *K. littledalei*, we used the RNA-seq data from nine species from *Kobresia* species. In total, 26,046 primitive gene models were predicted after integrating results of the three sources of evidence by EVM^21^. We then filtered and polished these gene models through expression level and evidence number, and 22,979 genes with FPKM > 1 or supported by more than two lines of evidence were retained (Table s6). For gene models only supported by one line of evidence, we searched SwissProt^22^, KEGG^23^, NCBI-nr, InterPro^24^ and Pfam for homologs. Gene models with homologs in any of the databases were retained, resulting in 157 genes. In total, 23,136 gene models were identified. The average length of genes and CDS are 3,545.25 bp and 1,163.41 bp, respectively, and there are 5.39 exons in each gene with length of 215.84 bp per exon (Table 1). Among them, 12,726 gene models are supported by all three lines of evidence (Fig. s2).

To assess the completeness of the gene identification, we conducted BUSCO analysis on 23,136 gene models. For 1,440 expected embryophyta genes, 86.2% complete and 3.6% fragmented gene models were identified in *K. littledalei*. The identified gene model was 89.8%, which is less than pearl millet (95.4%), broomcorn millet (98%), sugarcane (95.4%) and other recently published Poaceae species^25–27^. We also download the genome sequence of *A. comosus* and conducted BUSCO analysis; 92.6% complete and 2.7% fragmented gene models were identified^28^. To explore the reason, we also conducted BUSCO analysis on the transcriptomes (leaf) of *Kobresia tibetica, K. royleana* and *K. pygmaea* assembled by Trinity, and 81.4%, 74.6% and 79.3% complete gene models plus 4.1%, 5.6% and 6.0% fragmented gene models were identified, respectively (Table s7). Simultaneously, the transcriptomes of three other Cyperaceae species, *Cyperus papyrus* (shoot without flower), *Lepidosperma gibsonii* (leaves and buds) and *Mapania palustris* (leaf shoots) were downloaded from the 1,000 plants (1KP)^29^ project. The complete gene models were 57.1%, 64.7% and 39.5% and the fragmented gene models were 14.4%, 13.3% and 23.3% assessed by BUSCO analysis (Table s7).

Functional annotation of protein coding genes was obtained by mapping protein sequences to SwissProt^22^, KEGG^23^ and NCBI-nr protein databases by BLASTP to get the best hit. Simultaneously, functional annotation of protein coding genes was inferred by protein domains identified by searching the protein sequence against the InterPro^24^ and Pfam^30^ databases using InterProScan^31^ and HMMER^32^. The Gene Ontology (GO)^33^ terms were obtained by Blast2GO^34^. A total of 22,892 (98.95) out of 23,136 genes have integrated functional annotation (Table s8).

### Genome structure of *K. littledalei*

**Fig. 3:**
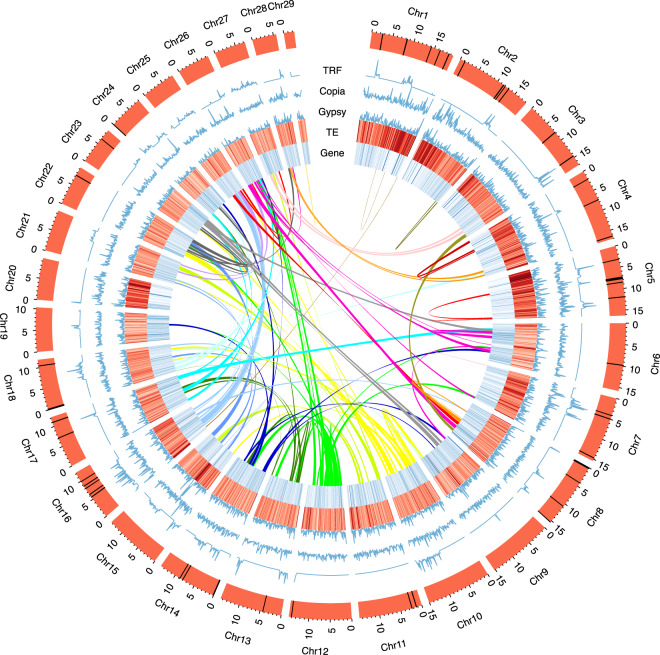
Features of the *Kobresia littledalei* genome.

The genus *Kobresia*, which includes about 70 species, is distributed predominantly in the alpine mountains of the Northern Hemisphere, and a majority of the 59 species found throughout China live on the Qinghai-Tibet Plateau. The basic chromosome numbers of species in *Kobresia* vary a lot (x = 16, x = 26, x = 29 and so on), which indicates that great changes have occurred in the chromosome structure of *Kobresia*. Moreover, it is reported that more than one-half of the tested species are polyploid in *Kobresia*, which is high compared with 5.7% in the closely related genus *Carex*^35^. The changes of chromosome structure and polyploidization in Kobresia likely indicate how these species adapted to the harsh environment of the Qinghai-Tibetan Plateau. The length of chromosomes of *K. littledalei* ranges from ~2.46 M to ~19.67 M. The centromeric regions were identified using an approach described by Robert *et al*.^36^. The base centromere repeat was 162 bp and highly abundant tandem repeats were identified on 18 chromosomes. The highly abundant tandem repeats dispersed on chromosome with high TE density like Chr1, Chr2, Chr5 and Chr16 (Fig. 3).

### Evolutionary and comparative genomic analysis

**Fig. 4:**
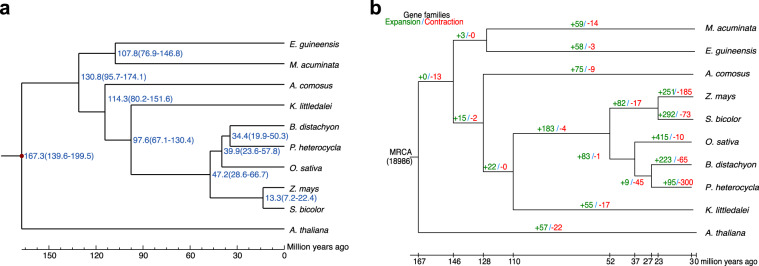
The phylogenetic relationships and divergence times of commelinid plants, and contraction and expansion of gene families. (**a**) The phylogenetic relationships and divergence times of commelinid plants. Phylogenetic reconstructions using concatenation of 1,077 genes and the maximum likelihood (ML) method with *A. thaliana* as the distant outgroup. Divergence times were estimated using the ‘mcmctree’ program incorporated in the PAML package. (**b**) Contraction and expansion of gene families. Numbers in green represent expanded families on this clade, and numbers in red represent contracted families on this clade.

To explore the evolutionary relationship of *K. littledalei*, we used OrthoFinder^34^ to cluster its genes with those from eight other commelinid monocots: *B. distachyon*, *O. sativa*, *Z mays*, *Sorghum bicolor*, *A. comosus*, *Elaeis guineensis*, *Musa acuminata, Phyllostachys heterocycla* and one dicot *A. thaliana*. From these ten species, we identified 826 one-to-one single-copy genes that were used to construct a maximum likelihood (ML) tree to show the evolutionary relationships using RaxML with the GTRGAMMA model^37^. Divergence times were estimated using the ‘mcmctree’ program incorporated in the PAML package^38^. According to the phylogenetic tree, *K. littledalei* separated from Poaceae about 97.6 million years ago after separating from Bromeliaceae about 114.3 million years ago (Fig. 4a).

**Fig. 5:**
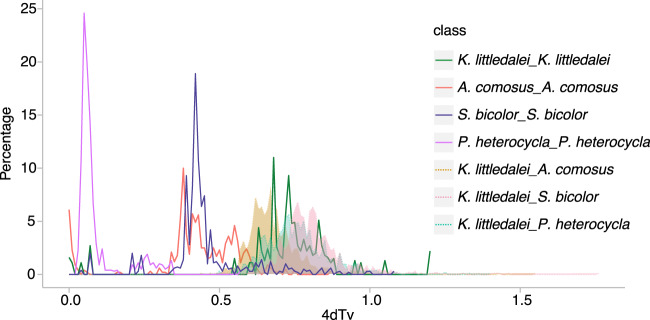
Whole genome duplication events in *Kobresia littledalei* and other Poaceae plants.

To clarify the genome duplication history of *K. littledalei*, we screened the paralogs within syntenic blocks of *K. littledalei* by McScan^39^ and calculated the distribution of the rate of transversions on fourfold degenerate synonymous sites (4DTv). There is one peak with values of 4DTv at 0.63–0.68, which indicated that one whole genome duplication (WGD) event occurred before the rho WGD event that occurred ~70 MYA in the grass lineage^40^. To investigate the genome duplication history in Poales, we also screened the orthologs with syntenic blocks between *K. littledalei* and *A. comosus, O. sativa* and *S. bicolor* separately. Simultaneously, we calculated the 4DTv of the paralogs in *A. comosus, O. sativa* and *S. bicolor*, which showed an obvious WGD with a 4DTv value of 0.4. The 4DTv peaks between *K. littledalei* and *A. comosus, O. sativa* and *S. bicolor* are between 0.6–0.8, near the WGD of *K. littledalei* but earlier than the WGD of *A. comosus*, *O. sativa* and *S. bicolor*. Taken together, Cyperaceae separated from Bromeliaceae and Poaceae during the time of the WGD of *K. littledalei* and they were subjected to WGD independently after differentiation (Fig. 5).

Comparing gene families among seven monocots, including *K. littledalei*, *O. sativa*, *S. bicolor*, *A. comosus*, *E. guineensis*, *M. acuminata and P. heterocycla*, we identified 23,136 *K. littledalei* genes in 12,006 families, with 8,645 gene families shared among them and 117 gene families unique in *K. littledalei* (Fig. s3). The expansion and contraction of the gene families, which can implicate the evolutionary dynamics of genes, were indicated by gene copy number in each family. In total, 17 gene families were contracted, and 55 gene families were expanded in *K. littledalei* (Fig. 4b). The expanded gene families included F-box containing protein, agamous-like MADS-box protein, and B3 domain containing protein (Table s9).

## Data Records

The raw data of the whole genome was submitted to the National Center for Biotechnology Information (NCBI) SRA with accession number SRP198441^41^. The final assembly and annotation had been deposited at GenBank SWLB00000000^42^. Gene functional annotations, repeat annotation and results of evolutionary analysis had been deposited at Figshare^43^.

## Technical Validation

### Evaluation of the genome assembly

For the final assembly, we used CEGMA^44^ to assess the completeness of the assembled *K. littledalei* genome, and 233 (93.95%) and nine (3,63%) genes out of 248 core eukaryotic genes had complete and partial alignment sequence in the assembly, respectively (Table s10). Of the 1,440 expected embryophyta genes, 84.5% were identified as having complete BUSCO profiles and 2.5% had fragmented BUSCO profiles of the 1440 expected embryophyta genes (Table s11). In total, 80.63% of the transcripts assembled by Trinity^45^ using *Kobresia* RNA-seq data covered 90% by one scaffold and 92.89% of transcripts covered 50% by one scaffold (Table s12). We evaluated the assembly continuity by analyzing the LTR Assembly Index (LAI)^46^, which is a standard method of assessing repeat sequences. The *K. littledalei* LAI score is 14.8, which indicated good continuity of the assembly.

For the preliminarily assembled sequences, we also used BUSCO version 3 (BUSCO, embryophyta odb9) assessing the completeness^47^, and 87.5% of the 1440 expected embryophyta genes were identified as having complete BUSCO profiles (Table s11). This result indicated that some real genome sequences were deleted during fusing of haplotype contigs. We compared the missing BUSCO profiles among *K. littledalei* genome assembly, transcriptomes of *Kobresia tibetica*, *Kobresia royleana, Kobresia pygmaea, Cyperus papyrus, Lepidosperma gibsonii* and *Mapania palustris*, 84 out of 181 were common in Cyperaceae species (Table s7, Fig. s4). This indicated that these genes are missing in all Cyperaceae species or varies a lot in Cyperaceae species compared to other embryophyta.

We used SOAPdenovo^48^ to assemble the unmapped Illumina reads to final assembly (Table s13), and 48,147,213 bp of sequence were assembled with N50 = 282 and longest_contig = 7749 (Table s14). We identified 2,274 genes (Supplementary set in Table s6) from the 48.15 M SOAPdenovo^48^ assembled sequences. The average gene length and the number and length of exons were all less than genes identified from the final assembly, resulting from the short length of the assembly (Table s6). After combining these sequences with the final assembly, 0.6% of the fragmented expected embryophyta genes increased.

These results indicated that some portion of our genome assembly is still missing. And the heterozygosity of the genome was evaluated to 1.68% from survey analysis (Table s2), which may increase the difficulty of the genome assembly.

## Supplementary information

Supplementary materials
