# Supplementary material for: Genome sequence of Kobresia littledalei, the first chromosome-level genome in the family Cyperaceae
Source: Sci Data. 2020 Jun 11;7:175. doi: 10.1038/s41597-020-0518-3 (PMC7289886; doi:10.1038/s41597-020-0518-3)
Supplement: Supplementary file 1 — Supplementary materials [file 41597_2020_518_MOESM1_ESM.pdf]

Table s1 Statistics of sequencing data....2
Table s2 Statistics of Survey....3
Table s3 Statistics of *Kobresia littledalei* preliminary genome assembly. 3
Table s4 Statistics of repetitive sequences of *Kobresia littledalei* genome. 3
Table s5 Statistics of TE classification....4
Table s6 Statistics of gene prediction....4
Table s7 Estimation of the completeness for gene identification based on BUSCO. 5
Table s8 Statistic of gene function prediction....5
Table s9 The gene number in each species and gene annotation of expanded gene families in *Kobresia littledalei*....6
Table s10 Estimation of the accuracy for *Kobresia littledalei* genome assembly based on CEGMA....9
Table s11 Estimation of the completeness for *Kobresia littledalei* genome assembly based on BUSCO....9
Table s12 Estimation of the completeness for *Kobresia littledalei* genome assembly based on mapping transcripts....9
Table s13 Statistics of mapping Illumina data to final assembly of *Kobresia littledalei*....9
Table s14 Statistics of assembly of unmapped Illumina reads....9
Figure s1 The K-mer distribution of Illumina paired-end reads.... 10
Figure s2 Statistics of predicted genes supported by three evidences. 11

Figure s3 Common and unique gene families compared among *Kobresia littledalei* (Klit), *Oryza sativa* (Osat), *Sorghum bicolor* (Sbic), *Phyllostachys heterocycla* (Phet), *Ananas comosus* (Acom), *Elaeis guineensis* (Egui), *Musa acuminata* (Macu). 12

Figure s4 Missing BUSCO profiles in Cyperaceae species including *Kobresia littledalei* (Klit), *Cyperus papyrus* (Cpap), *Kobresia pygmaea* (Kpyg), *Kobresia tibetica* (Ktib), *Kobresia royleana* (Kroy), *Mapania palustris* (Mpal) and *Lepidosperma gibsonii* (Lgib). 13

Table s1 Statistics of sequencing data.

Libraries	Insert size (bp)	Reads	Total data (G)	Read length (bp)	Sequence coverage (X)
Illumina reads	350	168,790,726	50.64	150	121.95
Pacbio reads	-	5,618,892	62.37	-	150.20
Hi-C	350	230,316,080	69.10	150	166.40
Total	-	-	182.11	-	438.55

Table s2 Statistics of Survey.

Kmer	Depth	N_kmer	Genome_size (M)	Revised genome size(M)	Heterozygous Rate (%)	Repeat rate (%)
17	96	40,541,402,288	422.31	415.24	1.68	53.93

Table s3 Statistics of *Kobresia littledalei* preliminary genome assembly.

Number of scaffolds	Total length of scaffolds (bp)	N50 of scaffolds (bp)	Longest scaffold (bp)	GC content (%)
1210	758,863,423	2,253,412	11,050,451	35.74

Table s4 Statistics of repetitive sequences of *Kobresia littledalei* genome.

Type	Repeat Size(bp)	% of genome
TRF	20,963,545	5.607895
RepeatMasker	193,263,347	51.6993
RepeatProteinMask	40,040,890	10.711219
Total	202,340,678	54.127549

Table s5 Statistics of TE classification.

	Denovo+Repbase		TE Proteins		Combined TEs	
	Length (bp)	%in Genome	Length (bp)	%in Genome	Length (bp)	%in Genome
DNA	66,592,519	17.81396	11,475,429	3.069758	69,259,079	18.52728
LINE	16,131,796	4.315368	7,381,234	1.974532	20,255,369	5.418453
SINE	359,441	0.096153	0	0	359,441	0.096153
LTR	103,666,160	27.73142	21,198,426	5.670728	104,166,188	27.86518
Unknown	12,807,908	3.426205	26,682	0.007138	12,834,590	3.433343
Total	193,263,347	51.6993	40,040,890	10.711219	197,921,429	52.94536

Table s6 Statistics of gene prediction.

Gene set		Number	Average gene length (bp)	Average CDS length (bp)	Average exons per gene	Average exon length (bp)	Average intron length (bp)
De novo	Augustus	25,005	2777.75	1089.9	4.76	228.95	448.84
	GlimmerHMM	40,920	7298.14	718.59	3.47	207.38	2669.1
	SNAP	48,194	4129.8	740.49	5.11	144.87	824.36
	Geneid	19,519	8626.73	880.74	5.72	153.95	1640.79
	Genscan	22,633	10018.75	1216.55	6.05	201.2	1744.27
Homolog	Zea_mays	24,936	2588.1	1206.3	3.73	323.61	506.59
	Arabidopsis_thaliana	26,399	2159.17	958.12	3.44	278.92	493.22
	Brachypodium_distachyon	23,821	2588.26	1261.74	3.92	321.71	453.97
	Ananas_comosus	22,686	3061.31	997.9	4.02	247.98	682.31
	Oryza_sativa	27,447	2255.06	1158.87	3.41	339.56	454.31
	Setaria_italica	22,745	2541.71	1018.56	4.01	254.04	506.14
RNA-seq	Cufflinks	61,391	5666.25	1261.3	4.98	253.2	1106.38
	PASA	23,969	3020.24	1009.08	5.08	198.61	492.83
EVM		26,046	3261.53	1088.86	4.98	218.75	546.22
PASA-update		26,046	3261.53	1088.86	4.98	218.75	546.22
Final set		23,136	3545.25	1163.41	5.39	215.84	542.54
Supplementary set*		2274	739.79	549.79	1.87	294.72	219.54

*These genes were identified from the sequence assembled from unmapped illumina reads.

Table s7 Estimation of the completeness for gene identification based on BUSCO.

Species	BUSCO notation assessment results
Kobresia littledalei C. B. Clarke genome genes	C:86.2% [S:67.6%, D:18.6%], F:3.6%, M:10.2%, n:1440
Transcriptome of *Kobresia tibetica*	C:81.4% [S:45.1%, D:36.3%], F:4.1%, M:14.5%, n:1440
Transcriptome of *Kobresia royleana*	C:74.6% [S:42.2%, D:32.4%], F:5.6%, M:19.8%, n:1440
Transcriptome of *Kobresia pygmaea*	C:79.3% [S:41.5%, D:37.8%], F:6.0%, M:14.7%, n:1440
Transcriptome of *Cyperus papyrus*	C:57.1% [S:53.6%, D:3.5%], F:14.4%, M:28.5%, n:1440
Transcriptome of *Lepidosperma gibsonii*	C:64.7% [S:57.5%, D:7.2%], F:13.3%, M:22.0%, n:1440
Transcriptome of *Mapania palustris*	C:39.5% [S:36.3%, D:3.2%], F:23.3%, M:37.2%, n:1440

*C：Complete BUSCOs, S：Complete and single-copy BUSCOs, D：Complete and duplicated BUSCOs, F：Fragmented BUSCOs, M：Missing BUSCOs, n：Total BUSCO groups searched

Table s8 Statistic of gene function prediction.

Database		Annotated Number	Annotated Percent (%)
NR		21,961	94.92
Swiss-Prot		17,822	77.03
KEGG		16,616	71.82
InterPro	Pfam	17,636	76.23
GO		16,122	69.68
Annotated		22,892	98.95
Total		23,136	-

Table s9 The gene number in each species and gene annotation of expanded gene families in *Kobresia littledalei*.

fam_id	Acom	Atha	Bdis	Egui	Kmyo	Macu	Osat	Phet	Sbic	Zmay	Annotation
OG0000005	0	0	23	0	45	0	58	13	43	3	F-box domain containing protein, expressed
OG0000013	6	0	17	20	25	7	36	4	19	12	F-box protein SKIP23-like
OG0000022	8	0	14	17	21	2	34	10	12	2	NA
OG0000043	3	28	3	3	46	2	3	3	4	2	pentatricopeptide repeat-containing protein
OG0000058	7	34	1	10	19	1	3	13	1	2	putative receptor-like protein kinase
OG0000104	1	0	2	7	50	1	4	5	2	2	F-box protein
OG0000134	2	5	2	5	15	11	9	2	11	4	S-norcoclaurine synthase 1-like
OG0000165	5	0	7	1	16	1	8	5	10	7	uncharacterized protein LOC100827825
OG0000174	13	1	3	2	22	4	9	1	2	2	UPF0481 protein
OG0000178	0	0	2	0	3	0	53	0	0	1	OSJNBa0021F22.8
OG0000179	43	0	3	0	9	0	1	2	0	0	uncharacterized protein LOC103632843
OG0000398	4	2	3	0	12	4	4	5	2	7	agamous-like MADS-box protein AGL14-like
OG0000404	1	1	1	6	11	3	6	4	7	3	UDP-glycosyltransferase 92A1-like
OG0000471	3	2	2	7	12	1	4	0	8	2	putative laccase-9
OG0000494	1	0	10	1	7	1	11	3	4	2	uncharacterized protein LOC105034708
OG0000498	10	1	2	0	22	0	3	1	1	0	F-box family protein
OG0000511	4	3	3	0	16	0	5	2	5	2	B3 domain-containing protein
OG0000552	0	1	3	3	7	0	12	1	5	7	hypothetical protein SORBIDRAFT_10g003830
OG0000592	1	30	1	0	5	0	1	0	0	0	uncharacterized protein LOC101312630
OG0000638	8	0	2	4	7	0	7	2	5	2	putative B3 domain-containing protein
OG0000662	2	0	2	7	12	2	2	2	5	2	probable aldo-keto reductase 1
OG0000706	1	1	4	6	7	2	8	2	3	2	uncharacterized protein LOC103983073

OG0000786	3	10	1	3	10	1	2	0	4	1	putative fatty acyl-CoA reductase 4
OG0000789	0	11	0	3	16	0	4	0	1	0	F-box protein family-like
OG0000795	0	0	2	0	21	7	1	2	1	1	Putative disease resistance protein RGA1
OG0000904	12	0	4	0	16	0	1	0	0	0	uncharacterized protein LOC102717464
OG0000953	0	0	2	1	7	3	13	2	4	1	hypothetical protein F775_05008
OG0000955	0	0	0	1	32	0	0	0	0	0	NA
OG0001152	0	0	0	0	30	0	0	0	1	0	hypothetical protein SORBIDRAFT_06g019220
OG0001282	24	0	0	0	5	0	0	0	0	0	uncharacterized protein LOC104908104
OG0001519	1	0	1	1	6	12	2	0	4	1	dirigent protein 1-like
OG0001522	1	1	6	2	6	1	1	2	7	1	uncharacterized protein LOC103999462
OG0001568	1	0	0	0	25	1	0	0	0	0	uncharacterized protein LOC104585233
OG0001810	0	0	1	0	7	0	11	1	2	4	NA
OG0001813	0	0	0	0	25	0	0	1	0	0	NA
OG0001994	0	2	1	2	14	0	3	1	1	1	F-box/FBD/LRR-repeat protein At1g13570-like
OG0002194	1	1	0	2	15	2	2	0	1	0	F-box protein At5g49610-like
OG0002230	0	0	6	0	5	2	1	3	2	5	mediator-associated protein 1-like
OG0002746	0	0	2	0	16	0	2	0	1	1	hypothetical protein OsJ_01660
OG0003033	0	0	1	0	19	0	1	0	0	0	hypothetical protein SORBIDRAFT_1138s002030
OG0003737	0	0	4	1	5	5	1	2	0	1	NA
OG0004073	1	0	3	1	3	0	0	0	5	5	F-box/FBD/LRR-repeat protein At1g13570-like
OG0004143	0	0	3	3	6	1	3	0	1	1	F-box protein At2g17036-like
OG0004144	0	0	0	0	9	1	1	3	0	4	pleiotropic drug resistance protein 3-like
OG0004145	0	0	0	0	8	2	2	4	1	1	Pleiotropic drug resistance protein 4
OG0004627	0	0	1	0	16	0	0	0	0	0	hypothetical protein SORBIDRAFT_1368s002010
OG0004631	0	0	0	0	17	0	0	0	0	0	uncharacterized protein LOC104090575 isoform X1

OG0004632	0	0	0	0	17	0	0	0	0	0	uncharacterized protein LOC103648078 isoform X3
OG0005093	1	0	0	4	6	1	0	3	1	0	putative receptor-like protein kinase At3g47110 isoform X3
OG0005755	0	0	1	0	3	1	0	1	0	9	phosphofructokinase V11
OG0006548	0	0	0	0	14	0	0	0	0	0	hypothetical protein OsI_39228
OG0006549	0	0	0	0	13	0	0	0	1	0	hypothetical protein OsI_30849
OG0007537	0	0	0	0	12	0	0	0	1	0	Putative disease resistance protein RGA3
OG0011703	0	0	2	0	4	0	3	0	1	0	NA
OG0013165	0	0	0	0	3	0	5	0	0	0	5-3 exoribonuclease 4-like isoform X1

Table s10 Estimation of the accuracy for *Kobresia littledalei* genome assembly based on CEGMA.

species	complete		complete + partial	
	Protein number	%completeness	Protein number	%completeness
Preliminary assembly	237	95.56	243	97.98
Final assembly	224	90.32	233	93.95

Table s11 Estimation of the completeness for *Kobresia littledalei* genome assembly based on BUSCO.

Genome	BUSCO notation assessment results
Preliminary assembly	C*:87.5% [S*:19.9%, D*:67.6%], F*:1.7%, M*:10.8%, n*:1440
Final assembly	C*:84.5% [S*:75.9%, D*:8.6%], F*:2.3%, M*:13.2%, n*:1440

*C：Complete BUSCOs, S：Complete and single-copy BUSCOs, D：Complete and duplicated BUSCOs, F：Fragmented BUSCOs, M：Missing BUSCOs, n：Total BUSCO groups searched

Table s12 Estimation of the completeness for *Kobresia littledalei* genome assembly based on mapping transcripts.

Genome	Number	Total length (bp)	Bases covered by assembly (%)	Sequences covered by assembly (%)	with >90% sequence in one scaffold		with >50% sequence in one scaffold	
					Number	Percent	Number	Percent
Preliminary assembly	80,085	126,726,010	97.471	99.806	71,418	89.178	78,997	98.641
Final assembly	80,085	126,726,010	92.896	97.441	64,569	80.626	74,393	92.893

Table s13 Statistics of mapping Illumina data to final assembly of *Kobresia littledalei*.

		Percentage
Reads	Mapping rate (%)	93.11
Genome	Average sequencing depth	107.08
	Coverage (%)	93.99
	Coverage at least 4X (%)	91.57
	Coverage at least 10X (%)	89.63
	Coverage at least 20X (%)	87.44

Table s14 Statistics of assembly of unmapped Illumina reads.

Number of scaffolds	Total length of scaffolds (bp)	N50 of scaffolds (bp)	Longest scaffold (bp)	GC content (%)
188,167	48,147,213	282	7,749	36.50

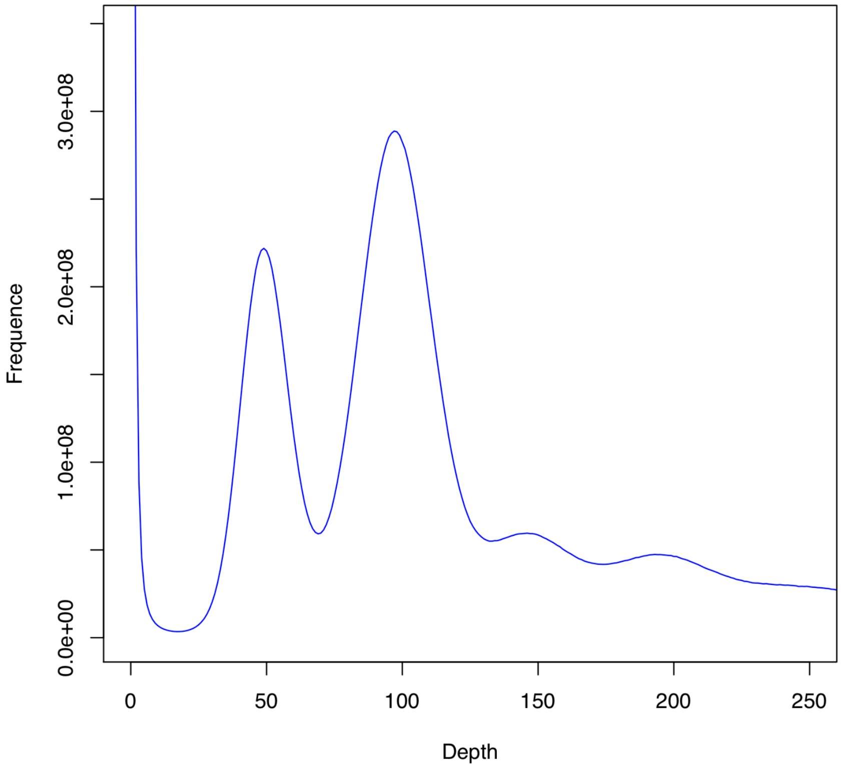

Figure s1 The K-mer distribution of Illumina paired-end reads.

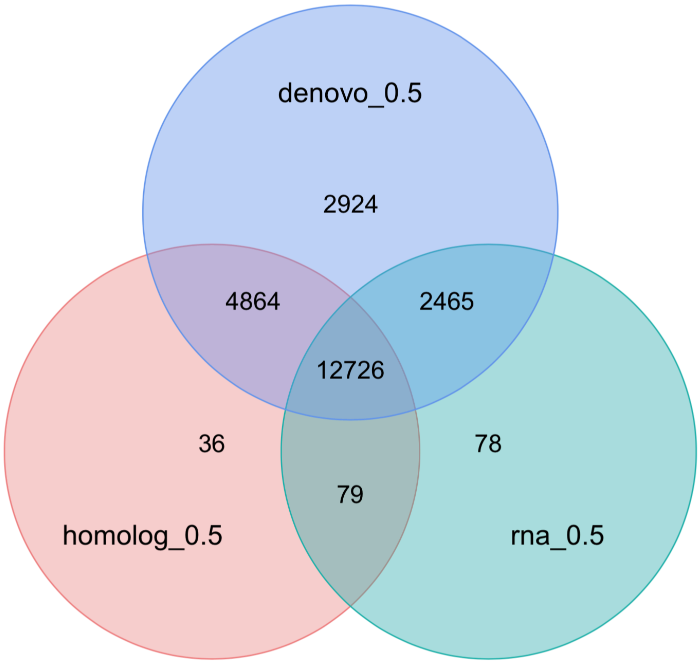

Figure s2 Statistics of predicted genes supported by three evidences.

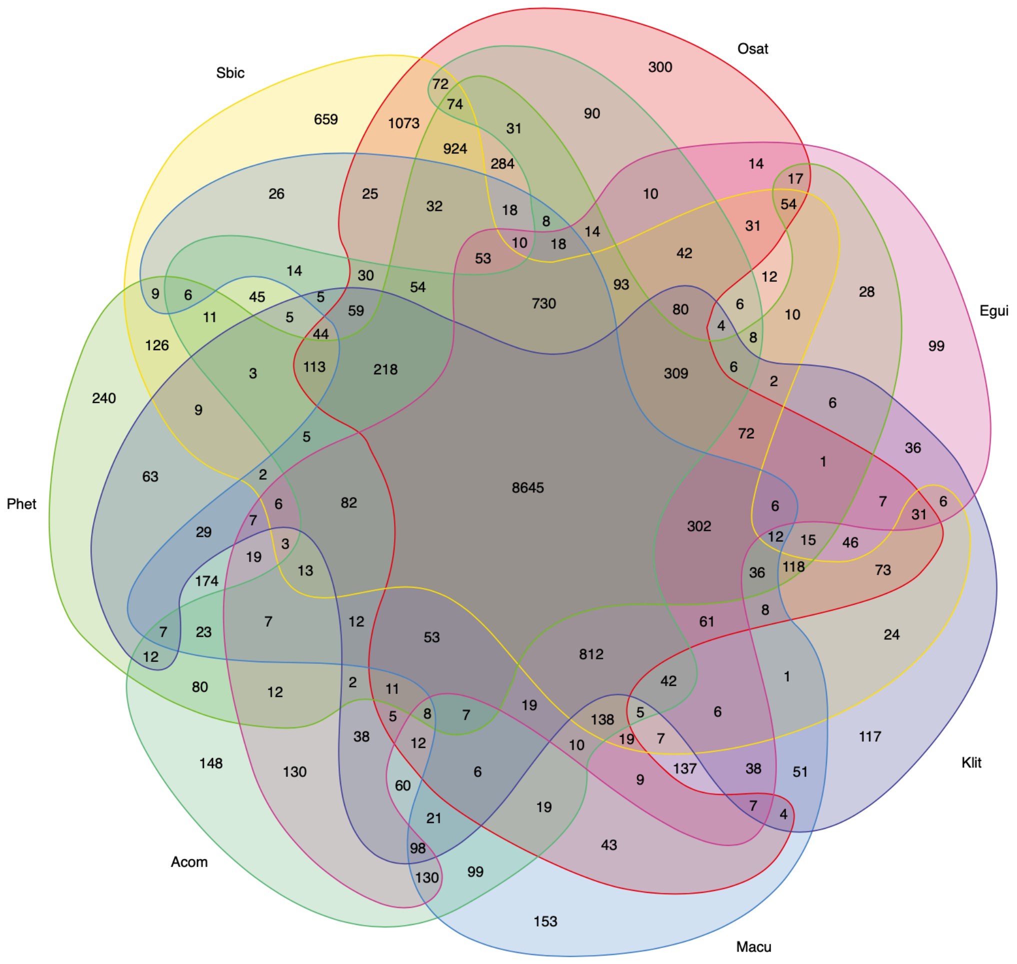

Figure s3 Common and unique gene families compared among *Kobresia littledalei* (Klit), *Oryza sativa* (Osat), *Sorghum bicolor* (Sbic), *Phyllostachys heterocycla* (Phet), *Ananas comosus* (Acom), *Elaeis guineensis* (Egui), *Musa acuminata* (Macu).

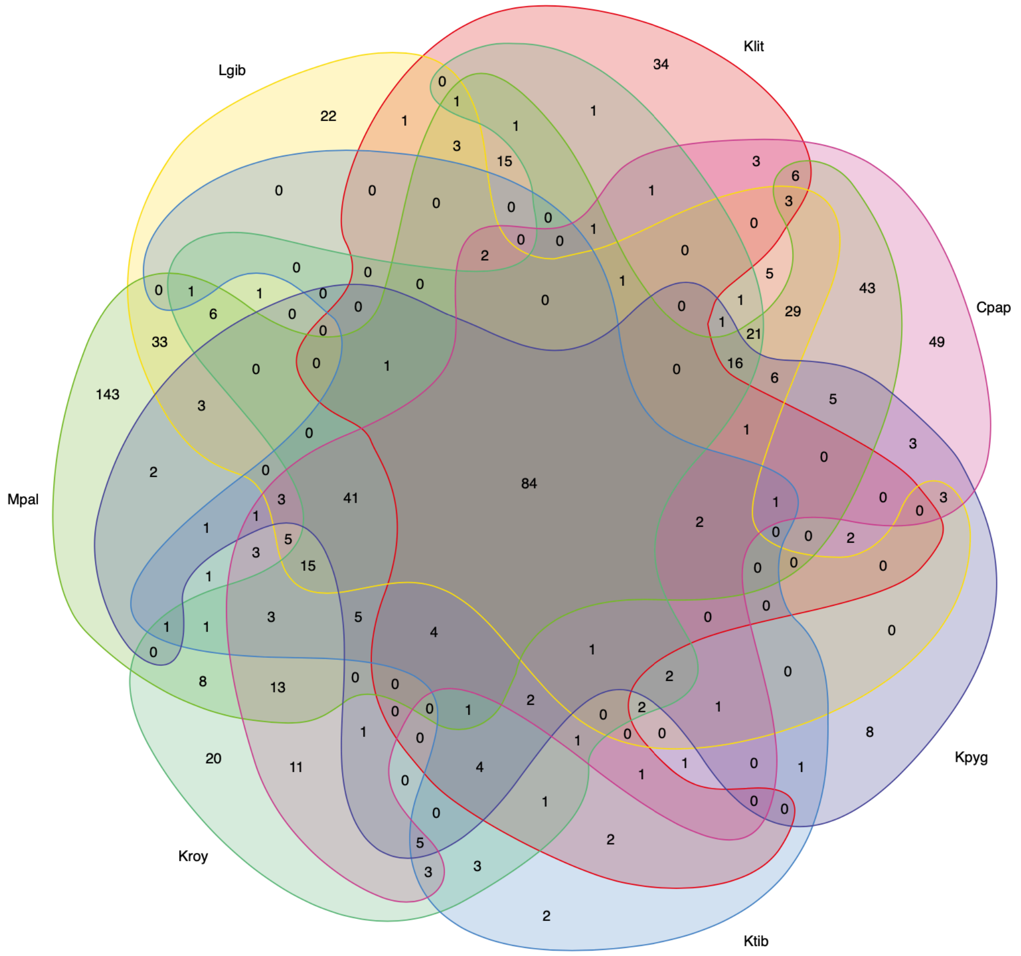

Figure s4 Missing BUSCO profiles in Cyperaceae species including *Kobresia littledalei* (Klit), *Cyperus papyrus* (Cpap), *Kobresia pygmaea* (Kpyg), *Kobresia tibetica* (Ktib), *Kobresia royleana* (Kroy), *Mapania palustris* (Mpal) and *Lepidosperma gibsonii* (Lgib).
